# Supplementary material for: Inference of hyperedges and overlapping communities in hypergraphs
Source: Nat Commun. 2022 Nov 24;13:7229. doi: 10.1038/s41467-022-34714-7 (PMC9700742; doi:10.1038/s41467-022-34714-7)
Supplement: Supplementary file 1 — Supplementary Information [file 41467_2022_34714_MOESM1_ESM.pdf]

Inference of hyperedges and overlapping communities in hypergraphs

Martina Contisciani,[1] Federico Battiston,[2] Caterina De Bacco[1,*]

[1] *Max Planck Institute for Intelligent Systems, Cyber Valley, 72076 Tübingen, Germany*

[2] *Department of Network and Data Science, Central European University, 1100 Vienna, Austria*

SUPPLEMENTARY NOTE 1. INFERENCE OF HYPERGRAPH-MT

A. Expectation-Maximization updates

The derivative in u_{ik} is given by:

$$\frac{\partial \mathcal{L}}{\partial u_{ik}} = - \sum_{e \in \Omega | i \in e} w_{d_e k} \prod_{j \in e | j \neq i} u_{jk} + \sum_{e \in \mathcal{E} | i \in e} A_e \frac{\rho_{ek}}{u_{ik}} \quad . \tag{1}$$

Setting this to zero, we obtain the updates:

$$u_{ik} = \frac{\sum_{e \in \mathcal{E} | i \in e} A_e \, \rho_{ek}}{\sum_{e \in \Omega | i \in e} w_{d_e k} \prod_{j \in e | j \neq i} u_{jk}} = \frac{\sum_{e \in \mathcal{E}} B_{ie} \, \rho_{ek}}{\sum_{e \in \Omega | i \in e} w_{d_e k} \prod_{j \in e | j \neq i} u_{jk}} \quad , \tag{2}$$

where B_{ie} is equal to the weight of the hyperedge e to which the node i belongs (it is an entry of the hypergraph incidence matrix). The numerator of Eq. (2) can be computed efficiently, as we only need the non-zero entries of the incidence matrix, which is typically sparse. Instead, computing the denominator can be prohibitive depending on the value of D, the maximum hyperedge size. This is due to the summation over all possible hyperedges in Ω, which requires extracting all possible combinations $\binom{N}{d}$, for $d = 2, \dots, D$. We propose a solution to this problem that reduces the computational complexity to $O(NDK)$. The key is to rewrite the summation over Ω such that we have an initial value that can be updated at cost $O(1)$ after one update of $u_{ik}^{(t)} \to u_{ik}^{(t+1)}$. Defining the set of hyperedges of fixed size d as $\Omega^d = \{e \in \Omega, d_e = d\}$ and $\bar{\Omega}^d_{ik} = \left\{e \in \Omega^d | i \notin e\right\}$, we can write more compactly:

$$u_{ik} = \frac{\sum_{e \in \mathcal{E}} B_{ie} \, \rho_{ek}}{\sum_{d=2}^{D} w_{dk} \sum_{e \in \bar{\Omega}^{d-1}_{ik}} \prod_{j \in e} u_{jk}} \quad . \tag{3}$$

The idea now is to observe that products like $\sum_{e \in \bar{\Omega}^{d-1}_{ik}} \prod_{j \in e} u_{jk}$ can be written as a function of $\sum_{e \in \Omega^{d-1}} \prod_{j \in e} u_{jk}$ and u_{ik}. The first term depends exclusively on d and k, not on a particular i. Hence, by isolating these terms from the ones that depend on u_{ik}, we only need to update the second terms, without re-computing the first. For instance, for $d = 3$, we can write $\sum_{e \in \bar{\Omega}^2_{ik}} \prod_{j \in e} u_{jk} = \sum_{j \neq m | j, m \neq i} u_{jk} u_{mk} = \sum_{e \in \Omega^2} \prod_{j \in e} u_{jk} - u_{ik} \sum_{j \neq i} u_{jk}$.

To formalize this, we define a function $\psi(S, k)$ that depends on a set of hyperedges S and community index k as

$$\psi(S, k) = \sum_{e \in S} \prod_{j \in e} u_{jk} \quad . \tag{4}$$

With this definition, we have

$$\sum_{e \in \Omega^d} \prod_{j \in e} u_{jk} = \psi(\Omega^d, k) = u_{ik} \, \psi(\bar{\Omega}^{d-1}_{ik}, k) + \psi(\bar{\Omega}^d_{ik}, k) \, , \tag{5}$$

valid for $d = 1, \dots, D$ and we fix the term $\psi(\bar{\Omega}^0_{ik}, k) = 1$. Notice that $\psi(\Omega^d, k)$ depends solely on d and k, and it can be used to compute $\psi(\bar{\Omega}^d_{ik}, k)$, needed in the denominator of Eq. (3). The advantage of using this formulation is given by the efficient update procedure. Indeed, when we update an entry $u_{ik}^{(t)} \to u_{ik}^{(t+1)}$ we can efficiently update

* caterina.debacco@tuebingen.mpg.de

$\psi^{(t)}(\Omega^d,k)$ by simply:

$$\psi^{(t)}(\Omega^d,k) = \left(u_{ik}^{(t)} - u_{ik}^{(t-1)}\right)\psi^{(t-1)}(\bar{\Omega}_{ik}^{d-1},k) + \psi^{(t-1)}(\Omega^d,k)\,, \tag{6}$$

and we can do this in parallel over $k=1,\dots,K$. These new values can then be used in the denominator of any other update $u_{jk}^{(t-1)} \to u_{jk}^{(t)}$ as

$$\psi^{(t)}(\bar{\Omega}_{jk}^{d},k) = \psi^{(t)}(\Omega^d,k) - u_{jk}^{(t-1)}\,\psi^{(t)}(\bar{\Omega}_{jk}^{d-1},k) \quad . \tag{7}$$

In practice, one only needs to initialize the values of $\psi^{(0)}(\Omega^d,k)$ at $t=0$ and then keep iterating in this way. There are $D\times K$ terms $\psi(\Omega^d,k)$ to compute at each update, costing $O(1)$ each. We have to repeat this N times (once after the update of each $\boldsymbol{u_i}=(u_{i1},\dots,u_{iK})$) for a total complexity of $N\times D\times K$. We have a similar complexity for updating the terms $\psi(\bar{\Omega}_{ik}^d,k)$. As for the initialization of $\psi^{(0)}(\Omega^d,k)$, at $t=0$ we assume a fixed $u_{ik}=u_k$ for each node and calculate Eq. (5) analytically. Note that one can then randomly initialize the u_{ik} and continue iterating using the updates above. We get

$$\psi^{(0)}(\Omega^d,k) = \sum_{e\in\Omega^d}\prod_{j\in e} u_k = \sum_{e\in\Omega^d} u_k^d = \binom{N_k}{d} u_k^d \quad , \tag{8}$$

where N_k is the number of nodes that have $u_{ik}>0$, i.e., the number of nodes initially in community k. With this formulation, the updates of the membership matrix become

$$u_{ik} = \frac{\sum_{e\in\mathcal{E}} B_{ie}\,\rho_{ek}}{\sum_{d=2}^{D} w_{dk}\psi(\bar{\Omega}_{ik}^{d-1},k)} \quad . \tag{9}$$

We now compute the derivative of $\mathcal{L}(\rho,\theta)$ in w_{dk}:

$$\frac{\partial\mathcal{L}}{\partial w_{dk}} = -\sum_{e\in\Omega^d}\prod_{j\in e} u_{jk} + \sum_{e\in\mathcal{E}|d_e=d} A_e\frac{\rho_{ek}}{w_{dk}} \quad . \tag{10}$$

Setting this to zero, we get the updates:

$$w_{dk} = \frac{\sum_{e\in\mathcal{E}|d_e=d} A_e\,\rho_{ek}}{\sum_{e\in\Omega^d}\prod_{j\in e} u_{jk}} = \frac{\sum_{e\in\mathcal{E}|d_e=d} A_e\,\rho_{ek}}{\psi(\Omega^d,k)} \quad , \tag{11}$$

which are computationally efficient and can be updated in parallel.

We describe the whole inference routine in Algorithm 1.

B. Priors, regularization, and constraints

So far, we infer the values of θ by following a maximum likelihood approach, which is equivalent to assuming uniform priors on the parameters. However, we can also posit non-uniform priors on the parameters and compute maximum a posteriori estimations. For instance, we may be interested in enforcing sparsity. To this aim, we can consider exponential distribution priors with parameters γ_u and γ_w for the parameters u and w, respectively. This results in two added terms in the log-likelihood, giving:

$$\mathcal{L}' = \mathcal{L} - \gamma_u\sum_{i,k} u_{ik} - \gamma_w\sum_{d,k} w_{dk} \quad . \tag{17}$$

Note, this is equivalent to a L_1-regularization on the values of u and w. Following the same computations as before, we get the new updates differ only by a constant term added in the denominators, e.g.,

$$w_{dk} = \frac{\sum_{e\in\mathcal{E}|d_e=d} A_e\,\rho_{ek}}{\gamma_w + \sum_{e\in\Omega^d}\prod_{j\in e} u_{jk}} \quad . \tag{18}$$

Similarly, we can arbitrarily add constraints on the parameters. For instance, we can impose the membership

Algorithm 1: Hypergraph-MT: EM algorithm

Input: hypergraph $\boldsymbol{A} = \{A_e\}_{e\in\mathcal{E}}$, number of communities K.

Output: membership matrix $\boldsymbol{u} = [u_{ik}]$; affinity matrix $\boldsymbol{w} = [w_{dk}]$.

Initialize $\boldsymbol{w}$ and $u_{ik} = u_k$ at random.

Compute

$$\psi^{(0)}(\Omega^d, k) = \binom{N_k}{d} u_k^d \quad , \tag{12}$$

Repeat until convergence:

1. Calculate $\boldsymbol{\rho}$ (E-step) for $k, e \in \mathcal{E}$:

$$\rho_{ek}^{(t)} = \frac{w_{d_e k}^{(t-1)} \prod_{i\in e} u_{ik}^{(t-1)}}{\sum_k w_{d_e k}^{(t-1)} \prod_{i\in e} u_{ik}^{(t-1)}}$$

2. Update parameters θ (M-step):

i) for each pair (d, k) update affinity matrix:

$$w_{dk}^{(t)} = \frac{\sum_{e\in\mathcal{E}|d_e=d} A_e \, \rho_{ek}^{(t)}}{\psi^{(t)}(\Omega^d, k)} \tag{13}$$

ii) for each pair (i, k):

ii.i) for each pair (d, k) update

$$\psi^{(t)}(\bar{\Omega}_{ik}^d, k) = \psi^{(t)}(\Omega^d, k) - u_{ik}^{(t-1)} \, \psi^{(t)}(\bar{\Omega}_{ik}^{d-1}, k) \tag{14}$$

ii.ii) update membership

$$u_{ik}^{(t)} = \frac{\sum_{e\in\mathcal{E}} B_{ie} \, \rho_{ek}^{(t)}}{\sum_{d=2}^{D} w_{dk}^{(t)} \, \psi^{(t)}(\bar{\Omega}_{ik}^{d-1}, k)} \tag{15}$$

ii.iii) for each pair (d, k) update

$$\psi^{(t+1)}(\Omega^d, k) = (u_{ik}^{(t)} - u_{ik}^{(t-1)})\psi^{(t)}(\bar{\Omega}_{ik}^{d-1}, k) + \psi^{(t)}(\Omega^d, k) \tag{16}$$

vectors to be probability vectors, i.e., $\sum_k u_{ik} = 1 \, \forall i$. Also in this case, it leads to a constant term added in the denominator of the updates of u_{ik}. In our numerical experiments, we run the model with and without constraints, and present the results of the model that performs the best.

SUPPLEMENTARY NOTE 2. EXPERIMENTS WITH SYNTHETIC DATA

The empirical data studied in the manuscript do not have ground truth labels, and therefore it is difficult to test the ability of the methods in recovering communities in hypergraphs. To this aim, we study the behavior of the models in synthetic data with known communities. We also use these data to provide a more precise estimate of the computational complexity of the methods. We generate hypergraphs by using the `dcsbm_hypergraph` function inside the package `xgi` (1), which generates synthetic hypergraphs by following a bipartite formalism. Notice that this data generating process differs from that of our generative model.

c_{in}/c_{out}	N	E	$\langle k \rangle$	s(k)	$\langle d \rangle$	s(d)	D	K
1	500	2230.7	44.6	24.2	10.0	5.4	29.7	3
2	500	2097.6	29.6	16.9	7.1	3.8	22.5	3
3	500	2013.4	24.4	13.7	6.1	3.1	19.1	3
4	500	1963.3	21.9	12.5	5.6	2.9	18.0	3
5	500	1911.9	20.3	11.6	5.3	2.7	17.2	3
6	500	1878.2	19.2	11.2	5.1	2.6	16.3	3
7	500	1866.1	18.7	11.0	5.0	2.5	16.2	3
8	500	1835.0	18.0	10.5	4.9	2.4	15.7	3
9	500	1820.3	17.5	10.4	4.8	2.4	14.8	3
10	500	1818.2	17.4	10.0	4.8	2.4	16.0	3

SUPPLEMENTARY TABLE I. **Summary of synthetic data with different strength of community structure.** Shown are the strength of the community structure (c_{in}/c_{out}), the number of nodes (N), number of hyperedges (E), mean node degree ($\langle k \rangle$), SD of node degree (s(k)), mean hyperedge size ($\langle d \rangle$), SD of hyperedge size (s(d)), maximum hyperedge size (D), and number of communities (K). The values are averages over ten independent samples.

A. Community detection

To test the ability of the methods in community detection in hypergraphs, we generate data with different values of assortative structure. In detail, we fix $N = 500$ nodes, $K = 3$ communities and approximately $E = 2000$ hyperedges. See Table I for a complete summary of the descriptive statistics of the data. In addition, this model takes in input an $\boldsymbol{\Omega}$ matrix that regulates the number of connections within (c_{in}) and between (c_{out}) communities, and we generate data by varying the strength of the community structure, measured by the ratio c_{in}/c_{out}. We fix $c_{in} = 2500$ and vary the ratio $c_{in}/c_{out} \in [1, \dots, 10]$, and for each value we generate ten independent samples. For this experiment, we use two additional methods for comparison: Hypergraph AON-MLL and Spectral Clustering. The first is the generative model proposed in (2), which generalizes the Louvain graph community detection method and assumes a symmetric partition function called All-Or-Nothing (AON) according to which edges are expected to lie fully within clusters. The second, instead, is the spectral method of (3) and performs community detection in hypergraphs by using the eigenpairs of the hypergraph Laplacian. More specifically, the communities are detected with the run of the k-means algorithm on the subspace spanned by these eigenvectors. Figure 1 shows results in terms of Normalized Mutual Information for the synthetic hypergraphs with three known communities. When $c_{in} = c_{out}$ the hypergraphs follow a random distribution and no community structure is induced; thus the communities are hard to detect. Conversely, the community structure becomes stronger as the ratio c_{in}/c_{out} increases, and it becomes easier to detect communities. Hypergraph-MT presents a clear pattern as its performance improves as the structure of the hypergraphs strengthens, and it significantly outperforms all the other methods, with the exception of Spectral Clustering, which however benefits from having an inference routine similar to the generative process of the synthetic data. Conversely, Graph-MT shows low and constant results regardless the strength of the community structure, which may get lost with the clique expansions. Instead, the curve of Hypergraph AON-MLL is flat around zero, illustrating the difficulty of the model to retrieve communities in synthetic data even when a strong assortativity is predominant. One explanation for its poor performance could rely on the assumptions behind the AON affinity function, which may be too strong and not appropriate to model this type of data. In fact, even though we have assortativity, this does not imply that *all* of the nodes in a given hyperedge are likely to be in the same community, but rather the majority of them. AON may be too sensitive to them being all in agreement. Perhaps other types of partition functions as described in (2) would be more appropriate, but they are also computationally prohibitive to run, thus making it impractical for our tests. In addition, Hypergraph AON-MLL benefits from having strong ground truth cluster signal in the low-size hyperedges (e.g., pairs or triangles) and may struggle in other cases. This could also be another possible explanation since the synthetic data have several hyperedges of higher size.

It is important to highlight that the synthetic data here used as ground truth were generated with an algorithm aimed at producing a planted partition from hard-membership communities (1). Indeed, both Hypergraph AON-MLL and Spectral Clustering are able to infer exclusively partitions where nodes are assigned to a single community. However, they lack the ability to capture the correct mesoscale organization of hypergraphs in more complex scenarios, where nodes can belong to more than one community at a time. To corroborate our statement, we have also investigated a different type of synthetic data, generated from a planted partition based on overlapping assignments of the nodes

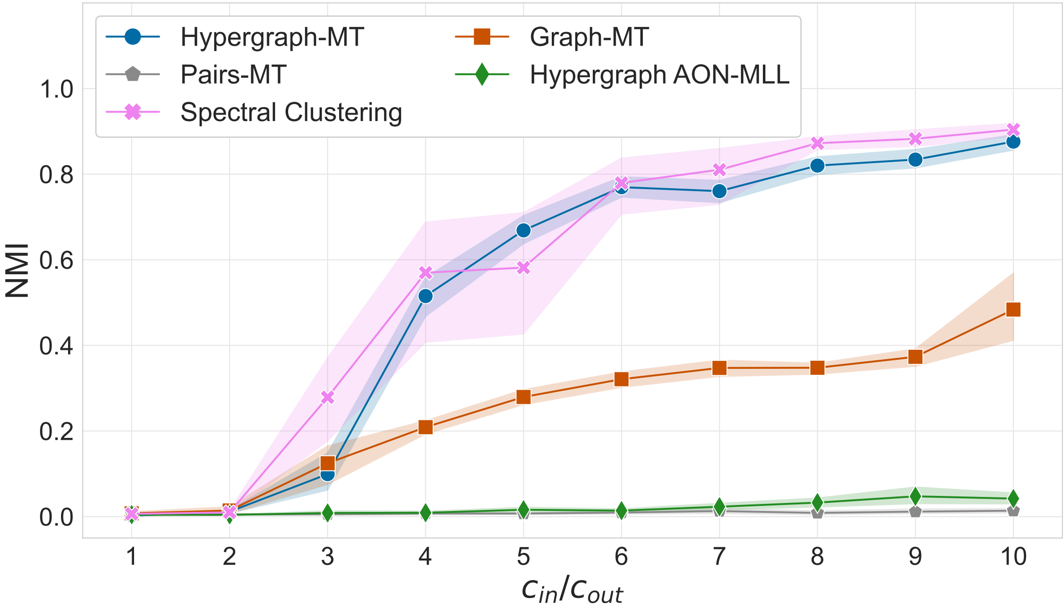

SUPPLEMENTARY FIGURE 1. **Community detection performance in synthetic hypergraphs with known communities.** We measure the Normalized Mutual Information in synthetic data generated by varying the strength of the community structure, measured by the ratio c_{in}/c_{out}. The descriptive statistics of the data are summarized in Table I, and the results are averages and standard deviations over ten independent samples. The plot shows how Hypergraph-MT and Spectral Clustering increase their performance as the community structure becomes stronger, while the other methods are not as robust. When a benchmark model with overlapping communities is used instead of one with hard communities, Hypergraph-MT clearly outperforms Spectral Clustering.

N	E	$\langle k \rangle$	s(k)	$\langle d \rangle$	s(d)	D	K
100	141.6	5.4	3.0	3.8	1.7	10.2	3
500	690.7	5.8	4.0	4.2	2.0	13.0	3
1000	1355.3	5.7	3.9	4.2	2.0	13.1	3
5000	6768.6	5.8	4.1	4.3	2.1	14.9	3
10000	13521.9	5.8	4.1	4.3	2.1	16.4	3

SUPPLEMENTARY TABLE II. **Summary of synthetic data with variable size.** Shown are the number of nodes (N), number of hyperedges (E), mean node degree ($\langle k \rangle$), SD of node degree (s(k)), mean hyperedge size ($\langle d \rangle$), SD of hyperedge size (s(d)), maximum hyperedge size (D), and number of communities (K). The values are averages over ten independent samples.

to multiple communities. If we consider a very simple model of overlapping community with two modules, where 25% of the nodes belong exclusively to the first community ($\boldsymbol{u} = [1, 0]$), 25% of the nodes belong exclusively to the second community ($\boldsymbol{u} = [0, 1]$), but 50% of the nodes participate with equal strength to the two communities ($\boldsymbol{u} = [0.5, 0.5]$), we find that Hypergraph-MT clearly outperforms Spectral Clustering. Indeed, comparing with ground truth, we obtain values of a cosine similarity CS = 0.97 for Hypergraph-MT and CS = 0.85 for Spectral Clustering. Moreover, the difference in performance between the two models becomes stronger when more complicated overlapping models are considered, even if we assign nodes predominantly to one community (a case which should favor hard-membership inference algorithms). For instance, if we consider a model where three groups of nodes of equal size are assigned community membership vectors of $\boldsymbol{u} = [0.55, 0.35, 0.1]$, $\boldsymbol{u} = [0.05, 0.6, 0.35]$ and $\boldsymbol{u} = [0.2, 0.2, 0.6]$ respectively, we obtain CS = 0.93 for Hypergraph-MT, but only CS = 0.67 for Spectral Clustering. A complete and detailed characterization of the hypergraph benchmark model used to generate ground truth data with overlapping communities will be provided in a separate manuscript currently in preparation.

B. Time complexity

In addition to the discussion in the manuscript, here we provide a proper assessment of the computational complexity of the various methods by running Hypergraph-MT, Graph-MT, and Pairs-MT on synthetic data with variable size. We generate hypergraphs with average node degree $\langle k \rangle \approx 6$, average hyperedge size $\langle d \rangle \approx 4$, and an $\boldsymbol{\Omega}$ matrix with entries $c_{in} = 2\,N$ and $c_{in}/c_{out} = 10$. We vary the number of nodes $N \in [100, 500, 1000, 5000, 10000]$, and we generate ten independent samples for each different value. See Table II for a complete summary of the descriptive statistics of the data. Figure 2 displays the running times of the algorithms for one iteration, and shows a good scaling with hypergraph's size N, with Hypergraph-MT being faster than Graph-MT across sizes. Notice that this computational complexity can be improved with a sparse implementation of the code, a task we leave for future work.

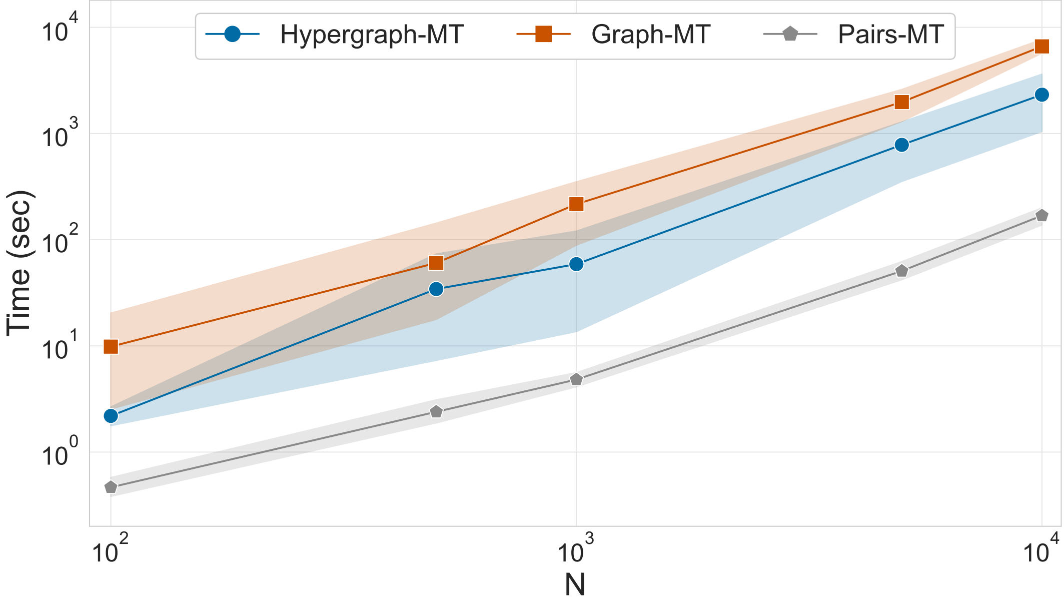

SUPPLEMENTARY FIGURE 2. **Computational complexity in synthetic data with variable size.** We measure the running time for one realization of the methods in synthetic data generated by varying the number of nodes. The descriptive statistics of the data are summarized in Table II, and the results are averages and standard deviations over ten independent samples. The plot shows a good scaling with hypergraph's size N, with Hypergraph-MT being faster than Graph-MT across sizes.

SUPPLEMENTARY NOTE 3. ANALYSIS OF THE GENE-DISEASE DATASET

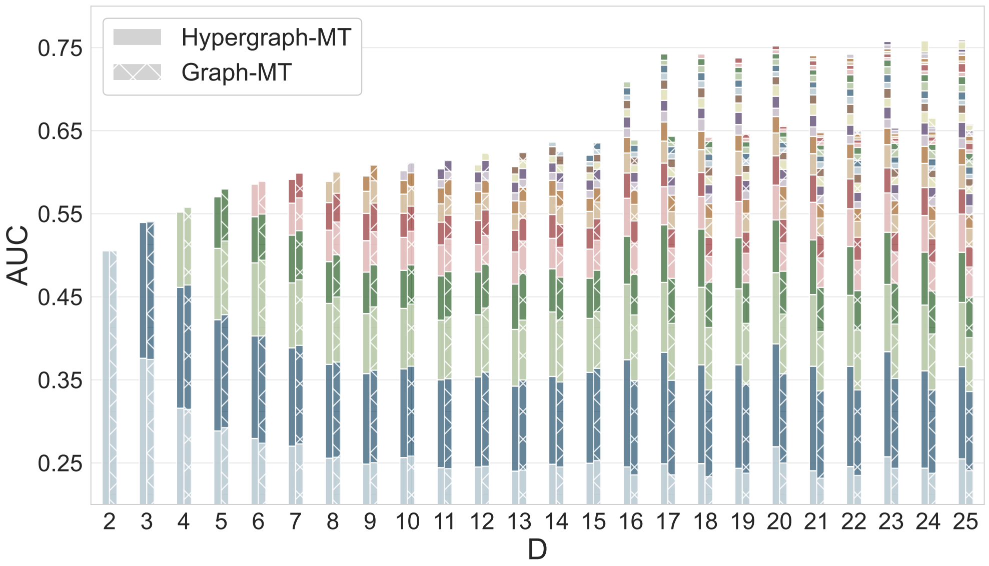

SUPPLEMENTARY FIGURE 3. **Cumulative hyperedge predictions in a Gene-Disease dataset.** We measure the AUC by varying the maximum hyperedge size D, and we plot the means over 5-fold cross-validation test sets. For each D, we show the cumulative performance for the different $2 \leq d \leq D$. The plot shows how the model on the hypergraphs (Hypergraph-MT) outperforms the one using the graphs obtained by clique expansions (Graph-MT) beyond the shift around $D = 15, 16$. In particular, Hypergraph-MT improves the predictive performance homogeneously across hyperedge sizes. Namely, it does not improve just in predicting the pairs-only, but also those of bigger sizes.

SUPPLEMENTARY REFERENCES

[1] `https://xgi.readthedocs.io/en/latest/api/generators/xgi.generators.nonuniform.html#module-xgi.generators.nonuniform`.

[2] P. S. Chodrow, N. Veldt, A. R. Benson, Generative hypergraph clustering: From blockmodels to modularity. *Science Advances* **7**, eabh1303 (2021).

[3] D. Zhou, J. Huang, B. Schölkopf, Learning with hypergraphs: Clustering, classification, and embedding. *Advances in neural information processing systems* **19** (2006).
